# The Case for a More Holistic Approach to Dry Eye Disease: Is It Time to Move beyond Antibiotics?

**DOI:** 10.3390/antibiotics8030088

**Published:** 2019-06-30

**Authors:** Azadeh Tavakoli, Judith Louise Flanagan

**Affiliations:** 1 School of Optometry and Vision Science, University of New South Wales, Sydney 2052, Australia; 2 Brien Holden Vision Institute, Sydney 2052, Australia

**Keywords:** Dry Eye Disease, microbiome, prebiotics, probiotics, functional foods

## Abstract

Dry eye disease (DED) is one of the most frequent presentations to optometrists with over 16 million US adults (6.8% of adult population) diagnosed as having this disorder. The majority of associated marketed products offer relief from symptomatology but do not address aetiology. DED harbours many distinguishing features of a chronic inflammatory disorder. The recent explosion in human microbiome research has sparked interest in the ocular microbiome and its role in the preservation and extension of ocular surface health and in the contribution of the gut microbiome to chronic systemic inflammation and associated “Western life-style” diseases. With a significant lack of success for many patients using currently available DED treatments, in this era of the microbiome, we are interested in exploring potential novel therapies that aim to reconstitute healthy bacterial communities both locally and distally (in the gut) as a treatment for DED. Although this direction of investigation is in its infancy, burgeoning interest makes such a review timely. This paper considers a number of studies into the use functional foods and associated products to ameliorate dry eye.

## 1. Introduction

Dry eye disease, a multifactorial disorder characterized by a loss of homeostasis of the tear film and hyperosmolarity, visual disturbance and ocular surface inflammation [1] encompasses ocular surface inflammatory disorders such as blepharitis (inflammation of the eyelids), ocular rosacea [2], and meibomian gland dysfunction (MGD) which is the most frequent cause of dry eye disease (DED) [3]. DED is an inflammatory disease that has many features in common with autoimmune disease [4]. The onset and progression of MGD is a vicious cycle of altered meibum consistency, bacterial proliferation on the eyelid, bacterial toxin production, fatty acid production, inflammation and tear film instability [3]. This cycle can be entered at different points. Exposure of ocular surface epithelium to hyperosmolarity contributes to the release inflammatory mediators [5], triggering further damage to epithelial cells, apoptosis, and goblet cell and glycocalyx mucin loss [6].

Altered immunity is a significant factor in DED. As articulated by Stern and colleagues [7], increasingly DED is recognized as a localized autoimmune disease driven by dysregulated immunoregulatory and inflammatory pathways of the ocular surface. Mucosal tolerance disruption is integral in the pathogenesis of DED [8], initiated when the immune balance of the ocular surface is altered due to internal or external factors. Stress to the ocular surface initiates a cascade of acute response cytokines and sequestering of auto response T cells that results in a chronic autoimmune response [7]. 

In addition to the localized autoimmunity, it has long been recognized that systemically delivered antibiotics can ameliorate ocular surface inflammation [9]. A number of antibiotics, both topical and oral, have been trialled for the treatment of MGD [10], blepharitis and (ocular) rosacea [11,12,13,14]. Complicating the situation however, a review by the American Academy of Ophthalmology into use of oral antibiotics for MGD-related ocular surface disorders [15] reported (despite “several review articles suggesting these agents as a mainstay of ocular surface disease care”) only scant level II and level III evidence to indicate efficacy while highlighting risk factors for systemic antibiotic use. Muddying the waters further are extant reports in the literature of germ-free mice and systemic antibiotic treatment animal models actually recapitulating DED rather than ameliorating it [16,17]. These diverse observations highlight the complex interplay of immunity, inflammation and the role of the ocular and gut microbiomes in the development, exacerbation or amelioration of DED. Although ocular surface disease is one of the most common reasons for visiting an ophthalmologist, it is currently considered largely incurable [6]. 

The purpose of this review is to explore the use of antibiotics in treating dry eye; as antimicrobial agents and/or anti-inflammatory mediators and to showcase the emerging field of DED treatment that seeks to redress the autoimmune dysfunction of DED through pro-biotics, pre-biotics and functional foods with a view to perhaps eventually moving beyond antimicrobial therapy with all its associated disadvantages.

Beyond antimicrobial agents, there are many over the counter treatments on the market for DED that are directed against symptoms, offering immediate but transient relief. A Cochrane review from 2016 studied 43 randomized trials (comprising 3497 dry eye subjects). The article was critical of the lack of homogeneity in variables such as diagnostic criteria, interventions, outcome measures and so forth, to the extent that the authors highlighted these constraints as potential confounders in their meta-analysis. Given these limitations (which illustrate a deficiency in many clinical trials for dry eye products) the authors concluded that overall there is little certainly that over-the-counter (OTC) drops offer any greater relief than placebo.

Dry eye ophthalmic drugs aim to provide relief in both signs and symptoms. A recent, elegant review by Holland and colleagues [18] provides a systematic analysis of topical ophthalmic drugs for treatment of dry eye that have been reported over the last twenty years. True to accepted dry eye wisdom, the authors report a lack of contemporaneous relief of signs and symptoms in any of the more than 100 studies reviewed. The review categorizes the various products into anti-inflammatories, mucin and tear secretagogues, and other investigational products. A lack of standardized testing is again highlighted in this investigation. 

Mucin secretogogues (that stimulate mucin or mucin-like substance production) appear to reduce corneal staining (which is suggestive of loss of corneal surface integrity) but attenuate few other signs of dry eye while (subjective) comfort appears to improve [19,20,21]. Mucin secretagogues along with hyaluronic acid are currently the mainstay of dry eye treatment in Japan [22]. However, common “treatment emergent adverse events” [18] include eye irritation, burning and stinging upon installation and eye itching. 

Biocidal action of antibiotics is seen as helpful in blepharitis- and MGD- induced dry eye through reduction of lid margin bacterial burden. Topical application of biocidal concentrations of fusidic acid and fluroquinolones have been advocated, with systemic cloxacillin and rifampin for recalcitrant disease, cautioned however by disadvantages of increasing antibiotic resistance [23].

Physical therapies aimed at stimulating meibum production (the theory of which is discussed at length by Borchman [24] and some novel experimental topical ophthalmic drugs [18] are further avenues of exploration. Overall however, lack of residence times, burning and stinging and eye irritation still trouble many of these products in addition to the discordance between improvement in signs and symptoms. 

The efficacy of corticosteroids in management of DED has been elucidated through molecular, cellular and clinical proofs of concept, illustrating the importance of inflammatory cascade in the progression of disease [25].

## 2. Dry Eye Disease as an Inflammatory Disorder

Increasing evidence reveals a model of dry eye as a localized mucosal autoimmune disease originating from an imbalance in the protective immunoregulatory and proinflammatory pathways of the ocular surface [7]. In a mouse DED model, desiccating stress is sufficient to induce acute response cytokines that further enhance pro-inflammatory cytokine/chemokine production, followed by activation of pathogenic Th1 and Th17 cell responses [26,27]. Autoreactive T cells traffic to the ocular surface tissues potentiating chronic autoimmune response and pathology [7]. Anti-inflammatories work to inhibit the hallmark cognate deleterious cascade of DED.

Eye drop preservative toxicity affects ocular mucosal tolerance causing dry eye [28]. In a mouse model, dry eye can be eliminated through restoration of immune function, suggesting again that DED arises from immune dysregulation [26]. 

As such, more promising DED agents are those directed towards reducing inflammation and restoring normal tear film though currently there is no specific immunotherapy for DED [29]. A 2017 review of cyclosporin A (CsA) for the treatment of chronic dry eye [30] indicated strong evidence of efficacy in treating signs but not symptoms. However, this conclusion is at odds with a 2019 review that suggested significant improvement only in corneal staining [18] and side-effects including significant ocular irritation. CsA highlights a number of ocular therapeutics issues such as retention time [30], burning and stinging and installation site irritation [31]. 

Interleukin -1 (IL-1) mediates the inflammatory cascade but products aimed at antagonizing IL-1 have had mixed results in clinical trials as reported by Holland and colleagues [18]. One promising inhibitor of the inflammatory cascade is Lifitegrast, the first dry eye product to target LFA-1 (a T-cell receptor that amplifies inflammatory pathways through cytokine release) [22]. Lifitegrast trials offer tantalizing evidence for achieving attenuation of both signs and symptoms. However, these milestones were not achieved in any single study but shown independently in separate studies [18]. This proved the test case for the FDA to forgo requiring the need for improvement against placebo in both signs and symptoms within the same study [32].

### 2.1. The Anti-Inflammatory Role of Antibiotics in DED

The use of systemic antibiotics for DED is well established. Anti-inflammatory effects of bacteriostatic tetracyclines such as oxytetracycline, doxycycline and minocycline are advocated for meibomian gland dysfunction induced DED though treatment breaks after three months are recommended [33]. Anti-inflammatory actions include the suppression of leucocyte migration [34] and nitric oxide and reactive oxygen species production [35].

Macrolides such as azithromycin and tacrolimus exert similar anti-inflammatory activities as evidenced in trials to treat blepharitis and MGD [25,36,37]. Tacrolimus stimulates T cell activation by potentially blocking cytokine receptor expression and has shown evidence of effecting improved tear stability and ocular surface status in DED subjects [38]. Azithromycin has also been reported to effect improvement in DED through direct action on meibomian gland epithelial cells by stimulating lipid accumulation and meibomian gland cell differentiation in vitro [39].

Additionally, low dose antimicrobials inhibit production of bacterial lipases and cognate free fatty acids that exacerbate inflammation, leading to improved meibomian gland function and stable tear film [40]. The release of toxic bacterial products such as lipases—or the secondary production and release of pro-inflammatory cytokines—is pathogenic [14]. Hence, in addition to anti-inflammatory and immunosuppressive action mediated through inhibition of lymphocyte activation and release of inflammatory mediators [41], low dose topical antibiotics have been used to inhibit production of bacterial toxins locally [42].

Even more so than biocidal use of antibiotics, such treatment is open to all the attendant disadvantages of chronic antibiotic use with low dosage more likely to lead to increased antibiotic resistance.

As useful as antibiotics, corticosteroids and other agents employed to modulate inflammation have been for DED, we need to move beyond merely keeping inflammation in check but rather, determining and ameliorating the source of inflammation [43].

### 2.2. The Case against Antibiotics for Dry Eye Disease

In relation to DED, antibiotics used to induce gut dysbiosis have resulted dry eye in experimental animal models [16]. Further, germ-free mice have been reported to spontaneously develop Sjögren syndrome-like inflammation and more severe dry eye than conventional mice [17]. Interestingly, restoration of the gut microbiome reversed the dry eye phenotype [17,44], suggesting compromised innate and adaptive effectors both at the ocular surface and in the gut contribute to increased susceptibility to DED [45]. Locally, a healthy ocular microbiome has been implicated in the homeostatic regulation of the ocular surface while antibiotic disruption of ocular bacterial homeostasis can initiate inflammation [46] and lead to potential deleterious perturbations of commensal microbiome that can cause undesirable side effects resulting from the collapse of the eye’s own immunodefense system [47].

But perhaps a greater issue to be addressed with all these products is the design concept of stimulating or initiating local responses for amelioration rather than addressing the aetiology of DED. As articulated by Goldstein and colleagues [48]: “DED remains a disorder of long-term maintenance rather than permanent cure” with current therapy addressing tear supplementation and localized transitory inhibition of inflammation. Since many patients with MGD suffer despite such treatment [16], how long lasting these treatments are, or whether they are establishing or further contributing to, the vicious cycle of DED [3] are important questions that need to be further addressed.

As has been established from the above, a large component of DED is inflammatory. Perhaps it is time we move beyond attempts to address only localized ocular inflammatory cascades and expand our search for a dry eye “cure” by consulting the chronic inflammatory disease literature. This review posits a case for a more holistic approach to combating signs and symptoms of DED.

### 2.3. The Anti-Inflammatory Role of Probiotics, Prebiotics and Funcational Foods in DED

Modern lifestyle choices appear to be an important factor in many inflammatory diseases, particularly those with an autoimmune or metabolic component including obesity, multiple sclerosis, rheumatoid arthritis, inflammatory bowel disease, type 1 diabetes, eczema, allergy, and psoriasis [20,49]. These illnesses comprise multifactorial aetiologies involving T-cell mediated autoimmune mechanisms leading to chronic conditions requiring ongoing disease management [49]. Regulatory and effector subsets of T cells are implicated in the pathogenesis of DED which is described as an inflammatory autoimmune disorder of the ocular surface [50] characterized by increased proinflammatory cytokines in the tears, epithelial apoptosis and leucocyte infiltration [8] and recapitulated by adoptive transfer of CD4^+^ T [51]. DED often requires life-long management and, as such, would appear well-placed within the family of chronic disease.

Hence, if we view DED as a localized autoimmune disease originating from an imbalance in the protective immunoregulatory and proinflammatory pathways of the ocular surface [52] we can draw from the burgeoning body of research into other chronic autoimmune disorders that are increasingly affecting the population.

Poor Western diets, typified by low fibre and high fat, have been shown to affect gut microbial composition and immune system functions and consequently trigger disease [53,54,55]. Gut dysbiosis contributes to a chronic inflammatory status and is a causative factor for various autoimmune disorders [56,57]. Thus, diet presents a vital route for immune system modulation [21,22,23,53,54,58].

Perturbation of the gut microbiome illustrated by germ-free or antibiotic-treated mice has demonstrated the regulation of lymphocytic development and inflammatory responses in the gut [59,60]. The effect of altered diet on gut microbiota in mice with systemic lupus erythematosus (SLE), an autoimmune disease mostly affecting skin and joints has been studied [56,61]. SLE innate inflammation caused an increase in the bacterial species *Lactobacillus reuteri* which, in turn, exacerbated systemic autoimmunity [62]. The authors reported a special starch diet decreased lupus development [62]. Such studies indicate that addressing gut dysbiosis for distal inflammatory disorders is a valid line of enquiry.

In an effort to address aetiology of DED, our group is interested in exploring alternative treatments for dry eye that re-establish a healthy ocular microbiome both through topical and systemic means; constraining ocular and gut dysbiosis and cognate local or systemic inflammation through the use of functional foods and related bioactive substances.

This article reviews a number of potential avenues of treatment for dry eye along the lines of functional foods and related products to work to effect healthier gut and ocular bacterial communities. Research in this arena in relation to dry eye is in its infancy but no doubt will be exploding in the next few years.

For this narrative review, a literature search was performed between January and May 2019 using electronic databases PubMed and Google Scholar. Included studies were clinical trials and meta-analyses and reviews of such, on the oral supplementation of probiotics, prebiotics and supplements in the form of functional foods for the treatment or prevention of DED and related disorders. Exclusion criteria compromised articles for which there was no full text available, articles written in a language other than English and grey literature. For inclusion into the review, as this is yet an emerging field of research, we identified 27 articles specific to this search from the first round and additional references were identified from related studies cited within these articles.

### 2.4. Potential Mechanism of Action of Prebiotics, Probiotics and Functional Foods in Addressing DED

Ocular surface pain and discomfort are prominent symptoms of chronic dry eye and as such, often provide the impetus for presentation to a specialist [7]. Conjunctival inflammation is a hallmark of all dry eye syndromes [63] with chronic inflammatory infiltration of the conjunctiva and lacrimal glands [5]. Factors associated with inflammatory pain, including pro-inflammatory cytokines and infiltrating inflammatory cells, are present in DED [7]. Interestingly, DED often co-presents in chronic inflammatory diseases such as irritable bowel syndrome and other disorders known to have a gut dysbiosis aetiology [64]. Other autoimmune disorders that share DED symptomatology include Sjögren syndrome, systemic lupus erythematosus, rheumatoid arthritis, thyroid disease, asthma, osteoarthritis, allergy and rosacea (though the autoimmune link with rosacea is still debated) [65,66]. Chronic inflammation is due in part to alterations in relative abundances between different bacterial phyla such as increases in Proteobacteria that leads to increased permeability of the gut and systemic host inflammation [67]. A similar dysbiosis has been reported in Sjögren syndrome [16].

In 2007 Graham and colleagues [68] compared the microbiome composition of the ocular surface in a group of subjects without dry eye to a group with DED and raised the question of whether resident bacteria were pathogens or commensals. They reported significant differences between the population and kind of bacterial species residing on the ocular surface of each group. Using both conventional culture and 16S rDNA they identified specific species including *Bacillus* spp. and *Klebsiella oxytoca*, as well as an overall increased bacterial count (CFU/swab) in participants with dry eye [68]. *Staphylococcus epidermidis* was present in 100% of samples. Later works have suggested that *S. epidermidis,* as an integral member of epithelial microflora, rather than merely providing a benign presence, might actually exert a probiotic function by preventing colonization of more pathogenic bacteria [69].

On the ocular surface, goblet cells are responsible for mucin production and hence reduction in these cells will reduce mucin production and disturb the healthy tear film. Graham et al. [68] showed increasing bacterial burden correlated with decreasing numbers of goblet cells. As a corollary, in a mouse model of irritable bowel syndrome in which mucosal inflammation was stimulated using normal colon microflora, a similar reduction in goblet cell depletion and inflammatory cell infiltration was noted [70]. It has been suggested that production of mucin on the ocular surface is analogous to production of glycoproteins in the gastrointestinal tract [71], similarly leading to the release particular glycans and polysaccharides as in the intestinal tract which boosts the growth of certain bacterial species [72].

There is precedence in the literature for the use of probiotic lysates, vitamins and omega-3 fatty acids to treat comorbid ocular, enteral and affective symptoms that comprise a disorder called “irritable eye syndrome” [73]. The authors of the study suggested that, via MALT (mucosal associated lymphoid tissue that is contiguous from the gut to the respiratory system to the naso-lacrimal system), subclinical inflammation arising from dysbiosis can cause or exacerbate signs and symptoms of DED since the eye contains its own local lymphoid tissues; the conjunctiva-associated lymphoid tissue (CALT), which samples antigens and maintains tolerance to commensal microbes [7]. Outcomes from the study indicated that supplements targeting gut dysbiosis might be a new approach to treating this syndrome.

## 3. Prebiotics

### 3.1. Definition

Prebiotics were defined about twenty years ago as “a non-digestible food ingredient that beneficially affects the host by selectively stimulating the growth and/or activity of one or a limited number of bacteria in the colon and thus improves host health” [74]. Throughout the last decade, in parallel with improved sequencing technologies leading to better understanding of the human microbiome, the concept of prebiotics has been modified several times. Finally, in 2016 the International Scientific Association for Probiotics and Prebiotics (ISAPP) published a consensus statement for prebiotics as “a substrate that is selectively utilised by host microorganisms conferring a health benefit” [75].

Antibiotics, minerals and vitamins can alter the gut bacterial composition but these are not considered as prebiotics. Prebiotics harbour distinct features. First, they are selectively utilised which means not utilised by the pathogenic microorganisms; second, evidence supports their beneficial effects on host; and third, the host enzymes should not degrade the compound [75].

Prebiotics alter the composition of the gut microflora and studies have shown the modification of faecal microbiota by adding non-digestible energy sources in the diet [76]. Clinical studies have demonstrated that the primary group of bacteria which are stimulated by prebiotics are Lactobacillus and Bifidobacterium both of which are commonly used as probiotics. Hence, prebiotics can contribute to the functional improvements of probiotics. It is also important to note that prebiotics do not influence pathogenic bacteria such as *Clostridia* and *Escherichia coli* [77,78,79]. 

Traditionally, non-digestive carbohydrates have been considered prebiotics and much research has focussed on three major groups; inulin, fructooligosaccharides (FOS) and galactooligosaccharides (GOS). According to the recent definition of prebiotics, particular non-carbohydrate compounds such as plant polyphenols may fit the prebiotic definition although more investigation needs to be done regarding this matter [75]. Further, Constantini and colleagues [80] make a convincing case for omega-3 polyunsaturated fatty acids (PUFA) to be considered prebiotics for their ability to modulate gut bacteria by supporting growth of lipoplysaccharide (LPS)-suppressing bacteria while concomitantly decreasing LPS-producing bacteria and *Enterobacteriaceae*.

### 3.2. Prebiotics and Human Health and Disease

In the last decade, evidence of prebiotics effect on the host-microbial community and the benefits for health has gained traction [81,82,83]. Because prebiotics are utilized by specific bacterial strains, this can elevate metabolism of select bacteria and their cognate metabolites without necessarily increasing the numbers of bacteria. In this way, other members of the community, as well as the host, can themselves benefit biologically or physiologically from prebiotics [84]. Physiological benefits of prebiotics include improvement of intestinal functions [85,86], increased mineral absorption (calcium) and improved bone density [87,88], regulation of lipid and glucose metabolism [79,89], and modulation of immune functions [79,90]. Moreover, it has been indicated that prebiotics can independently promote health, without necessarily influencing metabolism of beneficial bacteria; for example, they can bind with pathogens and create an anti-adherence effector they can improve the immune system by the effect on host immune receptors [91]. Intestinal leukocytes and specialized enterocytes sense the content of the intestinal lumen and influence the intestinal immune response. Oat β-glucan been shown to activate both cell types [92].

Plant oligosaccharides, flavonols and polyphenols confer numerous benefits including anti-oxidative and anti-inflammatory actions [93]. Often these agents are discussed in relation to their inflammatory or immune modulating functions without a complete understanding of the pathways involved. For example, the biovailability of such compounds has previously been seen as a disadvantage or a conundrum [94]. But now we are beginning to see that these compounds might be functioning as a food source for probiotic and indigenous bacteria both locally and through the gut and might even act locally as prebiotics on the ocular surface.

The impact of prebiotics on human health and different diseases has been explored in a number of studies. The efficiency of galactooligosaccharide (GOS) has been reported in irritable bowel syndrome (IBS) [95]. Also, Inulin and lactulose were examined in a study related to inflammatory bowel disease (IBD) [96]. Various studies have indicated a relationship between improvement of allergy conditions [96], skin health [96], metabolic disorders such as obesity [97].

Omega-3 fatty acids (FA) are essential polyunsaturated fatty acids obtained through diet. Short chain (alpha-linolenic acid) fatty acids are found in walnuts, soy, flaxseed and linseed, while long chain [eicosapentaenoic acid (EPA) and docosahexaenoic acid (DHA) subtypes are derived from oily fish or produced in the body from desaturation/elongation of short-chain FAs [98]. There is emerging evidence of their role as prebiotics. Omega-3 PUFAs have been shown in animal models to normalize early-life stress-induced gut dysbiosis [99] and, through the modulation of *Fimicutes/Baceroidetes* ratio, can reduce endotoxmeia associated with low grade inflammation—a distinguishing feature of chronic conditions including insulin resistance, obesity, IBD, anxiety, mood and depression, RA and cancer [80]. Extant reports suggest a reciprocal relationship between *Faecalibacterium* and *Bacteroidetes*. Additionally, omega-3 PUFAs can influence butyrate-producing genera such as *Bifidobacterium, Lachno-spira, Roseburia* and *Lactobacillus*, many of which are reported to be in dysbiosis in IBD subjects [80]. Omega-3 PUFAs are reported to effect increases in anti-inflammatory activity in the gut through increases short chain fatty acid-producing bacteria [100]. Animal studies suggest links between the Omega 3 PUFA-mediated alteration of the gut microbiome and positive immune impacts that together work to maintain gut mucosal integrity [80].

### 3.3. Prebiotics and Ophthalmic Related Diseases

Gut dysbiosis has been demonstrated in many inflammatory and autoimmune ocular diseases such as uveitis, which is an inflammatory condition of ocular tissues. A recent clinical study compared the gut microbiome of people with uveitis and control group, showing a reduction of gut microbiome diversity in people with uveitis. This reduction was shown to be more significant in anti-inflammatory bacterial strains [101].

Sjögren syndrome (SS) is an autoimmune disease with a variety of ocular complications. This disease affects lacrimal glands, the primary source of production of watery layer of the tear film and triggers DED and other ocular surface disorders. The microbiome shifts in SS have been previously reported, with suggestions for both prebiotic and probiotic interventions [102].

Apart from the extensive literature on omega-3 PUFAs in treatment of DED (in which studies the mechanism in not generally ascribed to be prebiotic), there are few reports addressing the use of prebiotics for DED. However, in a recent review of diet and rosacea (which included ocular symptoms) [103] a prebiotic diet was recommended.

In relation to dry eye, there has been robust investigation into the oral consumption of omega-3 fatty acids in the treatment of DED with mixed results initiated by the observation from the 32,000 participant Women Health study that drew an association between DED and low consumption of omega 3 PUFA [104]. By way of illustration of the controversy surrounding omega 3 PUFA and dry eye, a recent meta-analysis of omega 3 PUFA supplementation for DED, including results from randomized clinical trials comprising a total of 3363 subjects, concluded supplementation led to significant improvement in dry eye signs and symptoms [99], while the large multicentre NIH sponsored DREAM study, one of the most comprehensive so far, concluded there was no difference in dry eye outcome measures between test and placebo [105]. Both these and other studies report on the difficulties of direct comparison of results given variations in eligibility criteria, dose, placebo content, duration and so forth.

#### 3.3.1. Turmeric Extract and Curcumin

Turmeric, one of the most important herbal medicines in many Asian countries, is derived from Curcuma longa rhizome. Curcumin is a well characterized derivative of turmeric but fewer studies have explored the use of the more complex turmeric extract [106]. Turmeric extract can reach the colon without substantial digestion and is digested by, and in turn promotes, probiotic bacteria in the gut. Along with the presence of phenolic and flavonoid compounds, turmeric extract contains functional monosaccharides and polysaccharides that can reinforce the immune system [107]. A patented treatment for DED [108] mentions curcumin in the form of turmeric extract. In vitro evidence [109] suggests curcumin regulates hyperosmolarity-induced IL-1β, P38 and NF-κβ in human corneal epithelial cells (suggesting action additional to prebiotic functions) and modulates pro-inflammatory cytokine expression in an inflammatory conjunctiva mouse model [110].

#### 3.3.2. Fructo-Oligosaccharides

Based on the idea of the eye as an ecosystem in which inflammation and autoimmunity are linked to the microbiota, Chisari and colleagues [111] recruited 40 subjects for a randomized trial comparing a synbiotic mixture of probiotics and fructo-oligosaccharides with use of a tear substitute in reducing signs and symptoms of DED. After 30 days of treatment there were statistically significant improvements in Schirmer test (measuring tear secretion rate), tear break up time (a measure of tear film stability) and a reported “disappearance of symptoms” in addition to a reduction in *Staphylococcus aureus* colonization with a concomitant increase in *Staphylococcus epidermidis* colonization of the ocular surface.

The patent literature cites use of Aloe Vera and a prebiotic, fructan derived from chicory root, for amelioration of neurological symptoms, chronic pain, inflammatory bowel disease and dry eye, amongst other disorders [112]. Investigation into the use of Aloe Vera in ophthalmologic disorders is aimed at determining whether the rapid tissue repair and anti-inflammatory properties of Aloe Vera, which have been successfully demonstrated in the treatment of internal and external wounds, can be applied to disorders of the eye involving inflammation, ulceration and infections [112].

#### 3.3.3. Colostrum

Unique to milk are a structurally diverse group of oligosaccharides that rather than providing energy to the newborn, serve instead as prebiotics [113]. Back in 1994 colostrum was advocated as a cure for DED by Chaumeil and colleagues [114] who showed encouraging results in treating severe DED using enriched bovine colostrum. Bucolo and colleagues developed an atropine sulphate-induced dry eye model in rabbits to further explore this idea [115]. Comparing prebiotic properties of topical application of the milk oligosaccharide fucosyl-lactose to PBS following application of atropine sulphate to induce dry eye, the researchers noted significant improvement in tear stability measured by improved tear break up time and tear volume. Finally, a mouse study exploring the use of human milk in DED [116] reported both full cream and fat reduced milk were able to preserve corneal epithelial thickness in a preservative-induced dry eye mouse model with results comparable to that of cyclosporine.

#### 3.3.4. Quercetin

As a member of the flavonol class of flavonoids, quercetin is well known for its anti-oxidant and anti-inflammatory actions. Quercetin is found in high abundance in apples, grapes, onions, artichoke, fennel, celery, beans, chickpeas, plum, turnips, peppers, strawberries, tomatoes and broccoli [117]. However, quercetin is rapidly cleared following oral consumption and it has been suggested that it has low bioavailability [118]. Given that it has recently been suggested that quercetin, as well as other flavonoids, do not have to be absorbed in order to have an effect [118], the prebiotic effects of quercetin are now being explored in addition to direct anti-oxidative and anti-inflammatory activities properties with the prebiotic-like effect on the anti-inflammatory activity of certain Bifidobacterium species reported [93]. Use of quercetin has been explored in various ocular surface diseases [118] and found to provide immunoregulation when topically applied in mouse DED models. Abengózar-Vela and colleagues [119,120] have reported encouraging results in both a mouse model of DED and an in vitro model using human corneal epithelial cells. Topical quercetin results in increased tear volume, restoration of smooth corneal surfaces, increased goblet cell density and reduced ROS-induced pro-inflammatory gene expression [118]. Ho and colleagues report similar results with the use of quercetin in a desiccation stress DED mouse model with resulting increased tear volume, reduced corneal irregularities and increased goblet cells compared with control [121]. 

#### 3.3.5. Resveratrol

Similar to quercetin and curcumin, resveratrol has been shown in mouse models to act as a prebiotic, leading to increased levels of *Akkermansia* spp., bifidobacteria and lactobacilli [122]. Topical resveratrol has been shown, in isolation and in combination with quercetin, to decrease clinical signs of DED in an experimental mouse model of DED with improved corneal staining and similar anti-inflammatory effects to that of the prebiotic epigallocatechin gallate [120]. 

## 4. Probiotics

### 4.1. Definition

The term probiotic was introduced by Lilly and Stillwell in 1965 [123] as an agent conferring benefit for health existing in fermented milk [124]. The World Health Organization and the Food and Agriculture Organization of the United Nations define probiotics as “live microorganisms which when administered in adequate amounts confer a health benefit on the host” (FAO/WHO Working Group 2002). Generally, probiotic species belong to the genera *Lactobacillus* and *Bifidobacterium* with a few strains of other lactic acid bacteria, *Bacillus* spp., *Saccharomyces* spp. and *E. coli* [125,126,127]. Molecular-based techniques have however identified other genera and species including *Roseburia, Eubacterium* or *Faecalibacterium* spp. that might mitigate intestinal inflammation and enhance gut barrier defences [75,128]. Marketed probiotics can include different types of fermented or non-fermented food and added nutritional supplements in powder or tablet forms. They can also comprise single or multiple strains of bacteria [126,127].

### 4.2. Probiotics and Human Health and Disease

Probiotics and cognate products of metabolism effect equilibration of anti- and pro- inflammatory responses by various means [129]. Communication between the gut microbiome and innate and adaptive immune cells modulates immune tolerance and inflammation [129]. Probiotics have been trialled in treatment of irritable bowel syndrome (IBS) [130,131,132,133,134,135,136] and inflammatory bowel disease (IBD) and its subtypes ulcerative colitis (UC) [137,138], in addition to prophylactic treatment of atopic disease [139].

Epithelial cells protect the host from assault by pathogenic organisms and toxic agents by forming a mucosal barrier. Probiotics help maintain intestinal barrier integrity by competing with pathogens for nutrients and competing for attachment sites on epithelial cells, inhibiting attachment of pathogenic bacteria [129], and modulating the immune response, resulting in immunostimulation or immunoregulation. In addition, probiotics can reinforce the mucosal barrier defences by inducing antimicrobial peptides such as human defensins [140]. An extensive range of probiotics can suppress epithelial cell pro-inflammatory chemokine production following infection [141]. Daily consumption of yoghurt containing *Lactobacillus paracsei* spp., *paracasei*, *Bifidobacterium animalis* ssp., *Lactis* and heat-treated *Lactobacillus planatarum* has been shown to improve immune function [142]. In colitis and allergy, the efficacy of probiotics again suggests upregulation of anti-inflammatory cytokines while attenuating inflammatory cytokines [143]. Various strains of Lactic acid bacteria stimulate production of immunoglobulin A and cognate Ig-A secreting cells and as well interleukins 12 and 18 while reducing IgE synthesis and stabilizing mucosal barriers [144,145] thereby effecting an essential role in host mucosal protection against mucosal pathogens since IgA prohibits bacterial binding to epithelial cells and counteracts toxins.

Various probiotic strains reduce Th17 inflammatory cells production and expression of downstream cytokines TNF-α and INFγ [129] *L. rhamnosus* also suppresses Th2, TH17 and ameliorates clinical signs relation to atopic dermatitis, allergic asthma and rhinitis [146].

### 4.3. Probiotics and Ophthalmic Related Diseases

In relation to ocular uses of probiotics, a comprehensive systematic review and meta-analysis of allergic rhinitis conducted in 2016 considered ocular symptoms in twenty-two randomised, double-blind, placebo-controlled studies and reported an overall positive effect of probiotics in reduction of ocular symptom scores in allergic rhinitis [147].

Recently, Jhaveri Microbiology Centre of Brien Holden Eye Research Centre in India has examined the association between gut bacterial and fungal dysbiosis and bacterial keratitis. They analysed the bacterial and fungal composition of faecal samples of 21 healthy participants versus 19 patients with bacterial keratitis (BK). Anti-inflammatory bacteria including *Dialister*, *Megasphaera*, *Faecalibacterium*, *Lachnospira*, *Ruminococcus* and *Mitsuokella* and members of *Firmicutes*, *Veillonellaceae*, *Ruminococcaceae* and *Lachnospiraceae* were more evident in healthy individuals compared with people suffering from bacterial keratitis. They also found more pathogenic fungus in BK groups. Their result strongly suggests the imbalance of gut bacterial community in people with BK [148] and indicates that probiotics might help ameliorate BK.

Immune responses to commensal bacteria and opportunistic pathogens have been suggested as triggering Sjögren syndrome [149]. In a relatively recent clinical trial, the efficacy of Probiotic IRT-5 (consisting of *Lactobacillus casei*, *Lactobacillus acidophilus*, *Lactobacillus reuteri*, *Bifidobacterium bifidum* and *Streptococcus thermophiles*) on management of autoimmune uveitis (EAU) and autoimmune dry eye in mice was explored. Monitoring inflammatory score of retinal tissues for uveitis and corneal staining and tear secretion for dry eye, the authors report that probiotics can be effective in decreasing clinical signs of autoimmune dry eye [150]. Such data support the hypothesis that dry eye originates from an imbalance in the protective immunoregulatory and proinflammatory pathways of the ocular surface. Regulatory T cells on the ocular surface have been suggested to provide protection against autoimmunity, as demonstrated at other mucosal sites [151] and to protect mice exposed to desiccating stress from developing dry eye. When these cells are depleted, mice developed full blown disease [51]. It is speculated that inflammation in DED is due to a decrease in number and/or function of regulatory T cells and an increase in pro-inflammatory cells (CD4^+^ Th1/Th17) along with cognate inflammatory mediators [51]. As such, probiotics might well be considered as a potential therapy.

In 2016, a group of researchers examined the effect of a combination of probiotics plus vitamins in people with DED. Their results suggest that synbiotics, which combine probiotics and prebiotics in a form of synergism, can decrease some signs and symptoms of DED while also modulating gut function [152]. This group explored a combination of *Lactobacillus acidophilus*, *Streptococcus thermofhilus*, *Lactobacillus plantarum*, *Lactobacillus rhamnosus*, *Bifidobacteriumlactis*, Zn, Vitamins B1, B2, B6 and niacin.

Another dry eye study examining the effect of a combination of fish oil, lactoferrin, zinc, vitamin C, lutein, vitamin E, γ-aminobutanoic acid and *Enterococcus faecium* WB2000 on dry eye reported significant improvement in clinical symptoms at 4 and 8 weeks [153].

A 2017 report from Korea [150] explored the use of IRT-5 probiotics (*Lactobacillus casei, Lactobacillus acidophilus, Lactobacillus reuteri, Bifidobacterium bifidum and Streptococcus thermophiles*) on autoimmune dry eye. IRT-5 was efficacious in reducing dry eye through attenuation of autoreactive T cells, suggesting as the authors report, relevance to treatment of Sjögren syndrome with IRT-5. The working theory behind this research suggesting the severity of Sjögren syndrome is correlated with microbial dysbiosis [16], is that autoreactive T cells activated by peptides from oral, skin and gut bacteria activate autoreactive B cells leading to gut dysbiosis mediated increases in Th17 cells migrating into systemic circulation. Consequently, IRT-5 probiotics might suppress cross-reactive T cells against gut peptides resulting in decreased CD8^+^IFNγ^hi^ cells, leading to clinical improvement in SS.

In 2017, Chisari and colleagues [154] reported a pilot study into the aging eye microbiota in DED in patients treated with *Enterococcus faecium* and *Saccharomyces boulardii*. Their results showed reduction in disease severity through improvements in Schirmer tear test and tear film break up time in addition to alteration of the ocular microbiome.

Other studies have reported mixed results with use of probiotics with a recent systematic review reporting studies showing an increased risk of allergic conjunctivitis with administration of probiotics perinatally and other studies offering evidence for efficacy of probiotics [155].

## 5. Other Functional Foods

### 5.1. Red Ginseng

Many people who suffer from glaucoma and use associated medications complain of dry eye and have a higher prevalence of ocular surface disease than the general population [156,157]. The preservative within the drops (benzalkonuim chloride (BAK)) has been suggested as the cause of the reported associated inflammation, blepharitis and DED [158]. Red ginseng is a traditional folk remedy used in East Asia to treat a number of disorders including endocrine and immune disorders [158]. The chronic inflammatory association and goblet cells loss in DED are commonly treated with anti-inflammatory drugs. Bae and colleagues [158] report that ginsenoside within red ginseng possesses anti-inflammatory properties. Oral consumption of red ginseng was shown in two studies to improve signs and symptoms of DED. Both studies focused on dry eye in glaucoma patients [158,159]. In the earlier study by Kim it was noted as an aside to the main focus of the study (observations of ocular blood flow in glaucoma patients) that subjects reported ginseng relieved discomfort associated with use of anti-glaucoma drops and further, for some subjects, ginseng helped in reducing signs and symptoms of dry eye. Hence the latter study was designed to explore this earlier observation. As reported by the investigators, for those patients without full-blown glaucoma who are on anti-glaucoma medication, dry eye dominates their concerns [158]. The cause of dry eye in these subjects is multifactorial, however the preservative benzalkonium chloride (BAK) is strongly implicated. The authors reported improvements in subjective comfort rated by the ocular surface disease index, as well as increases in tear film breakup time, conjunctival hyperaemia and meibomian gland function with oral application of Korean red ginseng over 8 weeks. The authors drew parallels with omega-3 fatty acids. That red ginseng works to alleviate dry eye offers further support for the idea of immune-mediated systemic inflammation due to gut dysbiosis [158].

### 5.2. Honey

Honey features as a remedy in ancient civilisations, ancient Ayurvedic texts and teachings of Aristotle and Hippocrates as well as ancient Arab physicians [160]. Honey possesses a range of medicinal activities including anti-inflammatory, antibacterial, antifungal and wound healing [161]. A study of Hadza hunter-gatherers revealed a much more diverse gut microbiome compared to that of individuals from the industrialised West. Honey is plentiful at certain times in the Hazda diet and the collection and consumption of honey is associated with shifts in the gut microbiome [162]. More recent studies have shown honey also possesses anti-diabetic activity and positive oral health effects. Some of the oligosaccharides in honey are prebiotics [163]. Probiotic honey (honey enriched with probiotics) had a beneficial effect on marks of insulin metabolism and markers of inflammation in subjects with diabetic nephropathy [164]. For ocular use, Aristotle wrote of honey being a salve for sore eyes [165] along with ancient honey remedies for eye disease spans Attica, Europe, India, Asia and Africa [166]. Honey has been shown to be efficacious in treatment of endophthalmitis [167] and corneal oedema [151,168]. A report from Lithuania in 2007 showed improvement in DED with use of 20% honey solution eye drops [169]. There is also a growing body of work looking specifically at Manuka honey for use in Sjögren syndrome and non-Sjögren aqueous deficient dry eye and evaporative dry eye due to MGD [170].

### 5.3. Polyphenols

#### 5.3.1. Bilberry Extract

Anthocyanins, known collectively as flavonoids (part of the greatly diverse plant polyphenols) are the largest group of water-soluble pigments in the plant kingdom with reported protective effects including antioxidant, anti-allergic, anti-inflammatory, anti-viral, anti-proliferative and anti-microbial [171]. A 2017 study into bilberry extract and DED [172] reported significant improvement in tear secretion with suggestions of more pronounced effects with increased DED severity and improvements in biological antioxidant potential.

#### 5.3.2. Green Tea

Tea (from *Camellia sinensis)* is one of the most widely consumed drinks worldwide. Green tea contains numerous polyphenols (collectively, 30% dry weight) including low molecular weight phenols (such catechins), which account for approximately 25% of the dry weight of green tea [173] to which antioxidant, immunomodulatory [174] and antiviral activities have been ascribed [175]. In addition, green tea has beneficial effects against degenerative diseases, oxidative stress and chronic diseases [176], and lipid profile [177]. A 2017 double-blind placebo-controlled study into the efficacy of green tea in ameliorating dry eye and meibomian gland dysfunction due to its anti-oxidative, anti-bacterial, anti-androgen and immunomodulatory anti-inflammatory properties [174] reported significant improvement in TBUT, meibomian gland health and ocular comfort scores after one month. The authors suggest efficacy might be due to Epigallocatechin Gallate which exerts inhibitory effects on inflammation, through the suppression of IL-1, IL-6, MCP-1 and TNF-α and t NF-kB.

Contrary to this finding is a recent study reporting a single dose of green tea reduced tear quality as assessed by tear ferning patterns and phenol red thread test [173] postulated as being due to high polpyphenolic content leading to lipid oxidation and reduction in electrolyte concentration.

#### 5.3.3. Grape Polyphenols

A recent review into the anti-inflammatory activities of grape polyphenols (GPP), including grape derived proanthocyanidins, monomeric flavonoid, aglycones and related glycosides [178], suggested GPP to be of use for inflammation in blepharitis—both via direct antimicrobial action against various Gram positive and Gram negative bacteria [179], and via antagonism of pro-inflammatory enzymes such as cyclooxygenase, nitric oxide synthase, lipoxygenase and several inflammatory cytokines such as C-reactive protein, TNF-α, IL-1β and IL-6, NF-kB; and prostaglandins E2 and D2. Further evidence proffered [178] suggested GPP can reduce mylopen oxidase activity while grape derived delphinidin inhibits LPS-induced TNFα and IL-6 production. Grape antioxidants pycnogenol and resveratrol reduce inflammation of the eye and attenuate induction of pro-inflammatory markers such as IL-1α, IL-6 by scavenging intracellular ROS [178]. Hence these compounds are of interest in reducing ocular dysbiosis associated with blepharitis and DED.

### 5.4. Evening Primrose Oil

Oral evening primrose oil has been shown to reduce contact lens discomfort and associated dry eye [180]. The mechanism of action was hypothesized as dietary essential fatty acid supplementation regulating the inflammatory process. Dietary supplementation with the primrose oil-derived linoleic acid and gamma linolenic acid ameliorated symptoms and improved overall lens comfort in female patients with contact lens associated dry eye and additionally resulted in increased in tear production.

### 5.5. Glycerol Monolaurate

Through the increased intake in the modern Western diet over the last 50 years of partially hydrogenated vegetable oils and trans fatty acids, such as linoleic acid, there has been a concomitant loss of dietary lauric acids (such as glycerol monolaurate or monolaurin) [181]. This shift has set the stage for a pro-inflammatory state in the human body. Glycerol monolaurate (GML) is found in coconut and breast milk. It is known to inhibit exoprotein production by Gram positive bacteria including *S. aureus* [182]. In vitro studies have shown GML inhibits bacterial lipases (particularly *Staphylococcal* spp. that have been implicated in rosacea, blepharitis etc.) without disturbing the microbiota [183]. It is non-toxic and in low doses, not antimicrobial. It has the status of “Generally Recognized as Safe” by the FDA [183]. 

GML is used widely used in the food industry as a surfactant and preservative and this raises the possibility of ingested GML as a potential agent for reducing systemic inflammation, in addition to topical ocular application, given its good safety profile on skin and mucosal surfaces [165] and negative ocular irritation [184].

Previous studies conducted in our laboratory explored the efficacy of GML on inhibition of production of bacterial lipase from ocular isolates [40,183]. Further, we determined a range of concentrations for which GML inhibits lipase production without substantial antimicrobial action. Translation into clinical trials (topical and oral) will give us some indication as to whether such an approach can not only provide immediate relief through the anti-inflammatory properties of GML [185] but might produce a stable shift in the gut and/or ocular microbiome that results in a decreased bacterial lipase production through a natural rebalancing of a healthy ocular community as opposed to the action of antibiotics that eradicate commensal bacteria while limit toxin production by pathogens. We are interested in exploring both oral and topical GML for dry eye and initial trials are underway. Preliminary data indicate significant improvement with the use of a topical GML ointment compared to placebo over 6 weeks in reducing the severity of DED and associated signs including blepharitis, limbal conjunctival redness, conjunctival palpebral redness, meibomian gland expressibility and meibum quality. As with many other studies, there was a significant improvement in ocular comfort from baseline but this was also reported by users of the placebo.

### 5.6. Combination Nutraceuticals

A combination nutraceutical comprising fish proteins, rice carbohydrates, aloe Vera, papayam curcuma, green tea, omega3/6 fatty acids and other nturients has been used to treat dogs with keratoconjunctivitis sicca (KCS) [186]. Delivered for 60 days, this treatment showed improvement in Schirmer test, conjunctivitis and keratitis intensities and mucous “intensity” [186].

## 6. Conclusions

Development of novel therapies that provide a cure for dry eye is the holy grail of DED research. In rising to meet this challenge, perhaps we should revisit the very questions we are asking about DED to stimulate alternative hypotheses of disease aetiology that might give rise to rigorous, consistent, repeatable clinical research methodologies, effecting easier comparison of data. In this way we might even begin to close the treatment gap between signs and symptoms.

There is an emerging understanding of the efficacy of probiotics, prebiotics, synbiotics and other functional foods on gut and local ocular microbiota in modulation of ocular inflammation and ocular surface health and homeostasis. Just as antibiotics can have far reaching effects beyond purely antimicrobial efficacy, prebiotics, probiotics and functional foods can manifest their benefits in a number of ways. Re-envisioning the mechanism of action of these various agents which have featured strongly in the DED literature with often wildly differing outcomes, as agents that modify the microbiome, in addition to more direct effects, might focus clinical study design to tease out the various mechanisms of such agents and disambiguate extant data.

If DED is indeed on the spectrum of chronic inflammatory disorders, lifestyle changes along with medications aimed to reduce inflammation could be an effective strategy. Adherence to lifestyle changes for chronic disease languishes around 10% while medication adherence is around 50% [186]. As such, offering a “pill” for life-style associated chronic disorders appears the more effective route compared with dietary or physical activity interventions at the present time. Considering mechanisms of action derived from a microbiome-driven systemic or “holistic” approach to this disease might pave the way for a new generation of rigorous clinical studies to provide alternative and more effective solutions for management of DED.

## References

[1] Craig J.P., Nichols K.K., Akpek E.K., Caffery B., Dua H.S., Joo C.-K., Liu Z., Nelson J.D., Nichols J.J., Tsubota K. (2017). TFOS DEWS II Definition and Classification Report. Ocul. Surf..

[2] Lee S.H., Oh D.H., Jung J.Y., Kim J.C., Jeon C.O. (2012). Comparative Ocular Microbial Communities in Humans with and without Blepharitis. Investig. Ophthalmol. Vis. Sci..

[3] Baudouin C., Messmer E.M., Aragona P., Geerling G., Akova Y.A., Benítez-del-Castillo J., Boboridis K.G., Merayo-Lloves J., Rolando M., Labetoulle M. (2016). Revisiting the Vicious Circle of Dry Eye Disease: A Focus on The Pathophysiology of Meibomian Gland Dysfunction. Br. J. Ophthalmol..

[4] Messmer E.M. (2015). The Pathophysiology, Diagnosis, and Treatment of Dry Eye Disease. Dtsch. Arztebl. Int..

[5] Baudouin C. (2001). The Pathology of Dry Eye. Surv. Ophthalmol..

[6] Bron A.J., de Paiva C.S., Chauhan S.K., Bonini S., Gabison E.E., Jain S., Knop E., Markoulli M., Ogawa Y., Perez V. (2017). TFOS DEWS II Pathophysiology Report. Ocul. Surf..

[7] Stern M.E., Schaumburg C.S., Pflugfelder S.C. (2013). Dry Eye as a Mucosal Autoimmune Disease. Int. Rev. Immunol..

[8] Guzman M., Keitelman I., Sabbione F., Trevani A.S., Giordano M.N., Galletti J.G. (2016). Desiccating Stress-Induced Disruption of Ocular Surface Immune Tolerance Drives Dry Eye Disease. Clin. Exp. Immunol..

[9] Pflugfelder S.C. (2004). Antiinflammatory Therapy for Dry Eye. Am. J. Ophthalmol..

[10] Milner M.S., Beckman K.A., Luchs J.I., Allen Q.B., Awdeh R.M., Berdahl J., Boland T.S., Buznego C., Gira J.P., Goldberg D.F. (2017). Dysfunctional Tear Syndrome: Dry Eye Disease and Associated Tear Film Disorders-New Strategies for Diagnosis and Treatment. Curr. Opin. Ophthalmol..

[11] Barnhorst D.A., Foster J.A., Chern K.C., Meisler D.M. (1996). The Efficacy of Topical Metronidazole in the Treatment of Ocular Rosacea. Ophthalmology.

[12] Shine W.E., McCulley J.P., Pandya A.G. (2003). Minocycline Effect on Meibomian Gland Lipids in Meibomianitis Patients. Exp. Eye Res..

[13] Souchier M., Joffre C., Grégoire S., Brétillon L., Muselier A., Acar N., Beynat J., Bron A., D’Athis P., Creuzot-Garcher C. (2008). Changes in Meibomian Fatty Acids and Clinical Signs in Patients with Meibomian Gland Dysfunction after Minocycline Treatment. Br. J. Ophthalmol..

[14] Geerling G., Tauber J., Baudouin C., Goto E., Matsumoto Y., O’Brien T., Rolando M., Tsubota K., Nichols K.K. (2011). The International Workshop on Meibomian Gland Dysfunction: Report of the Subcommittee on Management and Treatment of Meibomian Gland Dysfunction. Investig. Ophthalmol. Vis. Sci..

[15] Wladis E.J., Bradley E.A., Bilyk J.R., Yen M.T., Mawn L.A. (2016). Oral Antibiotics for Meibomian Gland-Related Ocular Surface Disease: A Report by the American Academy of Ophthalmology. Ophthalmology.

[16] De Paiva C.S., Jones D.B., Stern M.E., Bian F., Moore Q.L., Corbiere S., Streckfus C.F., Hutchinson D.S., Ajami N.J., Petrosino J.F. (2016). Altered Mucosal Microbiome Diversity and Disease Severity in Sjögren Syndrome. Sci. Rep..

[17] Zaheer M., Wang C., Bian F., Yu Z., Hernandez H., de Souza R.G., Simmons K.T., Schady D., Swennes A.G., Pflugfelder S.C. (2018). Protective Role of Commensal Bacteria in Sjogren Syndrome. J. Autoimmun..

[18] Holland E.J., Darvish M., Nichols K.K., Jones L., Karpecki P.M. (2019). Efficacy of Topical Ophthalmic Drugs in the Treatment of Dry Eye Disease: A Systematic Literature Review. Ocul. Surf..

[19] Matsumoto Y., Ohashi Y., Watanabe H., Tsubota K., Group D.O.S.P.S. (2012). Efficacy and Safety of Diquafosol Ophthalmic Solution in Patients with Dry Eye Syndrome: A Japanese Phase 2 Clinical Trial. Ophthalmology.

[20] Takamura E., Tsubota K., Watanabe H., Ohashi Y. (2012). A Randomised, Double-Masked Comparison Study of Diquafosol Versus Sodium Hyaluronate Ophthalmic Solutions in Dry Eye Patients. Br. J. Ophthalmol..

[21] Tauber J., Davitt W., Bokosky J., Nichols K., Yerxa B., Schaberg A., LaVange L., Mills-Wilson M., Kellerman D. (2004). Double-Masked, Placebo-Controlled Safety and Efficacy Trial of Diquafosol Tetrasodium (Ins365) Ophthalmic Solution for the Treatment of Dry Eye. Cornea.

[22] Markoulli M., Hui A. (2019). Emerging Targets of Inflammation and Tear Secretion in Dry Eye Disease. Drug Discov. Today.

[23] Jackson W.B. (2008). Blepharitis: Current Strategies for Diagnosis and Management. Can. J. Ophthalmol..

[24] Borchman D. (2019). The Optimum Temperature for The Heat Therapy for Meibomian Gland Dysfunction. Ocul. Surf..

[25] Ganesalingam K., Ismail S., Sherwin T., Craig J.P. (2019). Molecular Evidence for The Role of Inflammation in Dry Eye Disease. Clin. Exp. Optom..

[26] Guzmán M., Sabbione F., Gabelloni M.L., Vanzulli S., Trevani A.S., Giordano M.N., Galletti J.G. (2014). Restoring Conjunctival Tolerance by Topical Nuclear Factor–κB Inhibitors Reduces Preservative-Facilitated Allergic Conjunctivitis in Mice. Clin. Exp. Optom..

[27] Stern M.E., Schaumburg C.S., Dana R., Calonge M., Niederkorn J.Y., Pflugfelder S.C. (2010). Autoimmunity at the Ocular Surface: Pathogenesis and Regulation. Mucosal Immunol..

[28] Galletti J.G., Gabelloni M.L., Morande P.E., Sabbione F., Vermeulen M.E., Trevani A.S., Giordano M.N. (2013). Benzalkonium Chloride Breaks Down Conjunctival Immunological Tolerance in a Murine Model. Mucosal Immunol..

[29] Farid M., Agrawal A., Fremgen D., Tao J., Chuyi H., Nesburn A.B., BenMohamed L. (2016). Age-related Defects in Ocular and Nasal Mucosal Immune System and the Immunopathology of Dry Eye Disease. Ocul. Immunol. Inflamm..

[30] Lallemand F., Schmitt M., Bourges J.-L., Gurny R., Benita S., Garrigue J.-S. (2017). Cyclosporine A Delivery to the Eye: A Comprehensive Review of Academic and Industrial Efforts. Eur. J. Pharm. Biopharm..

[31] Baudouin C., Figueiredo F.C., Messmer E.M., Ismail D., Amrane M., Garrigue J.-S., Bonini S., Leonardi A. (2017). A Randomized Study of the Efficacy and Safety of 0.1% Cyclosporine A Cationic Emulsion in Treatment of Moderate to Severe Dry Eye. Eur. J. Ophthalmol..

[32] Novack G.D. (2014). Why Aren’t There More Pharmacotherapies for Dry Eye?. Ocul. Surf..

[33] Bron A.J., Tiffany J.M. (2004). The Contribution of Meibomian Disease to Dry Eye. Ocul. Surf..

[34] Barabino S., Chen Y., Chauhan S., Dana R. (2012). Ocular Surface Immunity: Homeostatic Mechanisms and Their Disruption in Dry Eye Disease. Prog. Retin. Eye Res..

[35] Miyachi Y., Yoshioka A., Imamura S., Niwa Y. (1986). Effect of Antibiotics on the Generation of Reactive Oxygen Species. J. Investig. Dermatol..

[36] Foulks G.N., Borchman D., Yappert M., Kim S.H., Mckay J.W. (2010). Topical Azithromycin Therapy for Meibomian Gland Dysfunction: Clinical Response and Lipid Alterations. Cornea.

[37] Haque R.M., Torkildsen G.L., Brubaker K., Zink R.C., Kowalski R.P., Mah F.S., Pflugfelder S.C. (2010). Multicenter Open-Label Study Evaluating the Efficacy of Azithromycin Ophthalmic Solution 1% on the Signs and Symptoms of Subjects with Blepharitis. Cornea.

[38] Moscovici B.K., Holzchuh R., Chiacchio B.B., Santo R.M., Shimazaki J., Hida R.Y. (2012). Clinical Treatment of Dry Eye Using 0.03% Tacrolimus Eye Drops. Cornea.

[39] Liu Y., Kam W.R., Ding J., Sullivan D.A. (2014). Effect of Azithromycin on Lipid Accumulation in Immortalized Human Meibomian Gland Epithelial Cells. JAMA Ophthalmol..

[40] Flanagan J.L., Khandekar N., Zhu H., Watanabe K., Markoulli M., Flanagan J.T., Papas E. (2016). Glycerol Monolaurate Inhibits Lipase Production by Clinical Ocular Isolates without Affecting Bacterial Cell Viability. Investig. Ophthalmol. Vis. Sci..

[41] Hessen M., Akpek E.K. (2014). Dry Eye: An Inflammatory Ocular Disease. J. Ophthalmic Vis. Res..

[42] Federici T.J. (2011). The Non-Antibiotic Properties of Tetracyclines: Clinical Potential in Ophthalmic Disease. Pharmacol. Res..

[43] Jones L., Downie L.E., Korb D., Benitez-Del-Castillo J.M., Dana R., Deng S.X., Dong P.N., Geerling G., Hida R.Y., Liu Y. (2017). TFOS DEWS II Management and Therapy Report. Ocul. Surf..

[44] Wang C., Zaheer M., Bian F., Quach D., Swennes G.A., Britton A.R., Pflugfelder C.S., De Paiva S.C. (2018). Sjögren-Like Lacrimal Keratoconjunctivitis in Germ-Free Mice. Int. J. Mol. Sci..

[45] Kugadas A., Christiansen S.H., Sankaranarayanan S., Surana N.K., Gauguet S., Kunz R., Fichorova R., Vorup-Jensen T., Gadjeva M. (2016). Impact of Microbiota on Resistance to Ocular Pseudomonas Aeruginosa-Induced Keratitis. PLoS Pathog..

[46] Ozkan J., Willcox M.D. (2019). The Ocular Microbiome: Molecular Characterisation of a Unique and Low Microbial Environment. Curr. Eye Res..

[47] Vanderlaan D.G., Brown-Skrobot S.K., Schultz C.L. (1995). Ophthalmic Lens with Anti-Toxin Agent. Google Patents: 1995. U.S. Patent.

[48] Goldstein M.H., Rao N.K., Yanoff M., Duker J.S. (2018). Dry Eye Disease. Ophthalmology.

[49] Manzel A., Muller D.N., Hafler D.A., Erdman S.E., Linker R.A., Kleinewietfeld M. (2014). Role of “Western Diet” in Inflammatory Autoimmune Diseases. Curr. Allergy Asthma Rep..

[50] Chauhan S.K., El Annan J., Ecoiffier T., Goyal S., Zhang Q., Saban D.R., Dana R. (2009). Autoimmunity in Dry Eye Is due to Resistance of Th17 to Treg Suppression. J. Immunol..

[51] Niederkorn J.Y., Stern M.E., Pflugfelder S.C., De Paiva C.S., Corrales R.M., Gao J., Siemasko K. (2006). Desiccating Stress Induces T Cell-Mediated Sjögren’s Syndrome-Like Lacrimal Keratoconjunctivitis. J. Immunol..

[52] Yagci A., Gurdal C. (2014). The Role and Treatment of Inflammation in Dry Eye Disease. Int. Ophthalmol..

[53] Hooper L.V., Littman D.R., Macpherson A.J. (2012). Interactions between the Microbiota and the Immune System. Science.

[54] Kau A.L., Ahern P.P., Griffin N.W., Goodman A.L., Gordon J.I. (2011). Human Nutrition, the Gut Microbiome and the Immune System. Nature.

[55] Thorburn A.N., Macia L., Mackay C.R. (2014). Diet, Metabolites, and “Western-Lifestyle” Inflammatory Diseases. Immunity.

[56] Ruff W.E., Kriegel M.A. (2015). Autoimmune Host–Microbiota Interactions at Barrier Sites and Beyond. Trends Mol. Med..

[57] Yurkovetskiy L.A., Pickard J.M., Chervonsky A.V. (2015). Microbiota and Autoimmunity: Exploring New Avenues. Cell Host Microbe.

[58] Carmody R.N., Gerber G.K., Luevano J.M., Gatti D.M., Somes L., Svenson K.L., Turnbaugh P.J. (2015). Diet Dominates Host Genotype in Shaping the Murine Gut Microbiota. Cell Host Microbe.

[59] Buffie C.G., Pamer E.G. (2013). Microbiota-Mediated Colonization Resistance Against Intestinal Pathogens. Nat. Rev. Immunol..

[60] Lei Y.M.K., Nair L., Alegre M.-L. (2015). The Interplay Between the Intestinal Microbiota and the Immune System. Clin. Res. Hepatol. Gastroenterol..

[61] Engevik M.A., Ganesh B.P., Visuthranukul C., Versalovic J. (2017). Lactobacillus Reuteri Modulates Dendritic Cells and the Immune Response in Vitro and in Vivo. FASEB J..

[62] Zegarra-Ruiz D.F., El Beidaq A., Iñiguez A.J., Lubrano Di Ricco M., Manfredo Vieira S., Ruff W.E., Mubiru D., Fine R.L., Sterpka J., Greiling T.M. (2019). A Diet-Sensitive Commensal Lactobacillus Strain Mediates TLR7-Dependent Systemic Autoimmunity. Cell Host Microbe.

[63] Creuzot C., Passemard M., Viau S., Joffre C., Pouliquen P., Elena P.P., Bron A., Brignole F. (2006). Improvement of Dry Eye Symptoms with Polyunsaturated Fatty Acids. J. Fr. Ophtalmol..

[64] Galor A., Covington D., Levitt A.E., McManus K.T., Seiden B., Felix E.R., Kalangara J., Feuer W., Patin D.J., Martin E.R. (2016). Neuropathic Ocular Pain due to Dry Eye Is Associated with Multiple Comorbid Chronic Pain Syndromes. J. Pain.

[65] Matossian C., McDonald M., Donaldson K.E., Nichols K.K., MacIver S., Gupta P.K. (2019). Dry Eye Disease: Consideration for Women’s Health. J. Women’s Health.

[66] Vehof J., Kozareva D., Hysi P.G., Hammond C.J. (2014). Prevalence and Risk Factors of Dry Eye Disease in a British Female Cohort. Br. J. Ophthalmol..

[67] Tsai Y.-L., Lin T.-L., Chang C.-J., Wu T.-R., Lai W.-F., Lu C.-C., Lai H.-C. (2019). Probiotics, Prebiotics and Amelioration of Diseases. J. Biomed. Sci..

[68] Graham J.E., Moore J.E., Jiru X., Moore J.E., Goodall E.A., Dooley J.S.G., Hayes V.E.A., Dartt D.A., Downes C.S., Moore T.C.B. (2007). Ocular Pathogen or Commensal: A PCR-Based Study of Surface Bacterial Flora in Normal and Dry Eyes. Investig. Ophthalmol. Vis. Sci..

[69] Otto M. (2009). Staphylococcus Epidermidis—The ‘Accidental’ Pathogen. Nat. Rev. Microbiol..

[70] Fukushima K., Sasaki I., Ogawa H., Naito H., Funayama Y., Matsuno S. (1999). Colonization of Microflora in Mice: Mucosal Defense Against Luminal Bacteria. J. Gastroenterol..

[71] Koropatkin N.M., Cameron E.A., Martens E.C. (2012). How Glycan Metabolism Shapes the Human Gut Microbiota. Nat. Rev. Microbiol..

[72] Lu L.J., Liu J. (2016). Human Microbiota and Ophthalmic Disease. Yale J. Biol. Med..

[73] Feher J., Pinter E., Kovács I., Helyes Z., Kemény A., Markovics A., Plateroti R., Librando A., Cruciani F. (2014). Irritable Eye Syndrome: Neuroimmune Mechanisms and Benefits of Selected Nutrients. Ocul. Surf..

[74] Gibson G.R., Roberfroid M.B. (1995). Dietary Modulation of the Human Colonic Microbiota: Introducing the Concept of Prebiotics. J. Nutr..

[75] Gibson G.R., Hutkins R., Sanders M.E., Prescott S.L., Reimer R.A., Salminen S.J., Scott K., Stanton C., Swanson K.S., Cani P.D. (2017). Expert Consensus Document: The International Scientific Association for Probiotics and Prebiotics (ISAPP) Consensus Statement on the Definition and Scope of Prebiotics. Nat. Rev. Gastroenterol. Hepatol..

[76] Walter J., Ley R. (2011). The Human Gut Microbiome: Ecology and Recent Evolutionary Changes. Annu. Rev. Microbiol..

[77] Costabile A., Kolida S., Klinder A., Gietl E., Bäuerlein M., Frohberg C., Landschütze V., Gibson G.R. (2010). A Double-Blind, Placebo-Controlled, Cross-Over Study to Establish the Bifidogenic Effect of a Very-Long-Chain Inulin Extracted from Globe Artichoke (Cynara Scolymus) in Healthy Human Subjects. Br. J. Nutr..

[78] Depeint F., Tzortzis G., Vulevic J., I’Anson K., Gibson G.R. (2008). Prebiotic Evaluation of a Novel Galactooligosaccharide Mixture Produced by the Enzymatic Activity of Bifidobacterium Bifidum Ncimb 41171, in Healthy Humans: A Randomized, Double-Blind, Crossover, Placebo-Controlled Intervention Study. Am. J. Clin. Nutr..

[79] Roberfroid M., Gibson G.R., Hoyles L., McCartney A.L., Rastall R., Rowland I., Wolvers D., Watzl B., Szajewska H., Stahl B. (2010). Prebiotic Effects: Metabolic and Health Benefits. Br. J. Nutr..

[80] Costantini L., Molinari R., Farinon B., Merendino N. (2017). Impact of Omega-3 Fatty Acids on the Gut Microbiota. Int. J. Mol. Sci..

[81] Gibson G.R., Scott K.P., Rastall R.A., Tuohy K.M., Hotchkiss A., Dubert-Ferrandon A., Gareau M., Murphy E.F., Saulnier D., Loh G. (2010). Dietary Prebiotics: Current Status and New Definition. Food Sci. Technol. Bull. Funct. Foods.

[82] Hutkins R.W., Krumbeck J.A., Bindels L.B., Cani P.D., Fahey G., Goh Y.J., Hamaker B., Martens E.C., Mills D.A., Rastal R.A. (2016). Prebiotics: Why Definitions Matter. Curr. Opin. Biotechnol..

[83] Rastall R.A., Gibson G.R. (2015). Recent Developments in Prebiotics to Selectively Impact Beneficial Microbes and Promote Intestinal Health. Curr. Opin. Biotechnol..

[84] Louis P., Flint H.J., Michel C., Schwiertz A. (2016). How to Manipulate the Microbiota: Prebiotics. Microbiota of the Human Body: Implications in Health and Disease.

[85] Ashley C., Johnston W.H., Harris C.L., Stolz S.I., Wampler J.L., Berseth C.L. (2012). Growth and Tolerance of Infants Fed Formula Supplemented with Polydextrose (Pdx) and/or Galactooligosaccharides (Gos): Double-Blind, Randomized, Controlled Trial. Nutr. J..

[86] Tabbers M.M., Boluyt N., Berger M.Y., Benninga M.A. (2011). Nonpharmacologic Treatments for Childhood Constipation: Systematic Review. Pediatrics.

[87] van den Heuvel E.G.H.M., Schoterman M.H.C., Muijs T. (2000). Transgalactooligosaccharides Stimulate Calcium Absorption in Postmenopausal Women. J. Nutr..

[88] Weaver C.M., Martin B.R., Nakatsu C.H., Armstrong A.P., Clavijo A., McCabe L.D., McCabe G.P., Duignan S., Schoterman M.H.C., van den Heuvel E.G.H.M. (2011). Galactooligosaccharides Improve Mineral Absorption and Bone Properties in Growing Rats through Gut Fermentation. J. Agric. Food Chem..

[89] Alliet P., Scholtens P., Raes M., Hensen K., Jongen H., Rummens J.-L., Boehm G., Vandenplas Y. (2007). Effect of Prebiotic Galacto-Oligosaccharide, Long-Chain Fructo-Oligosaccharide Infant Formula on Serum Cholesterol and Triacylglycerol Levels. Nutrition.

[90] Jeurink P.V., van Esch B.C.A.M., Rijnierse A., Garssen J., Knippels L.M.J. (2013). Mechanisms Underlying Immune Effects of Dietary Oligosaccharides. Am. J. Clin. Nutr..

[91] Licht T.R., Ebersbach T., Frøkiær H. (2012). Prebiotics for Prevention of Gut Infections. Trends Food Sci. Technol..

[92] Volman J.J., Mensink R.P., Ramakers J.D., de Winther M.P., Carlsen H., Blomhoff R., Buurman W.A., Plat J. (2010). Dietary (1→3), (1→4)-β-D-Glucans from Oat Activate Nuclear Factor-κB in Intestinal Leukocytes and Enterocytes from Mice. Nutr. Res..

[93] Kawabata K., Sugiyama Y., Sakano T., Ohigashi H. (2013). Flavonols Enhanced Production of Anti-Inflammatory Substance(s) by Bifidobacterium Adolescentis: Prebiotic Actions of Galangin, Quercetin, and Fisetin. Biofactors.

[94] Anand P., Kunnumakkara A.B., Newman R.A., Aggarwal B.B. (2007). Bioavailability of Curcumin: Problems and Promises. Mol. Pharm..

[95] Silk D.B.A., Davis A., Vulevic J., Tzortzis G., Gibson G.R. (2009). Clinical Trial: The Effects of a Trans-Galactooligosaccharide Prebiotic on Faecal Microbiota and Symptoms in Irritable Bowel Syndrome. Aliment. Pharmacol. Ther..

[96] Ghouri Y.A., Richards D.M., Rahimi E.F., Krill J.T., Jelinek K.A., DuPont A.W. (2014). Systematic Review of Randomized Controlled Trials of Probiotics, Prebiotics, and Synbiotics in Inflammatory Bowel Disease. Clin. Exp. Gastroenterol..

[97] Dewulf E.M., Cani P.D., Claus S.P., Fuentes S., Puylaert P.G.B., Neyrinck A.M., Bindels L.B., de Vos W.M., Gibson G.R., Thissen J.-P. (2013). Insight into the Prebiotic Concept: Lessons from an Exploratory, Double Blind Intervention Study with Inulin-Type Fructans in Obese Women. Gut.

[98] Giannaccare G., Pellegrini M., Sebastiani S., Bernabei F., Roda M., Taroni L., Versura P., Campos E.C. (2019). Efficacy of Omega-3 Fatty Acid Supplementation for Treatment of Dry Eye Disease: A Meta-Analysis of Randomized Clinical Trials. Cornea.

[99] Pusceddu M.M., El Aidy S., Crispie F., O’Sullivan O., Cotter P., Stanton C., Kelly P., Cryan J.F., Dinan T.G. (2015). N-3 Polyunsaturated Fatty Acids (PUFAs) Reverse the Impact of Early-Life Stress on the Gut Microbiota. PLoS ONE.

[100] Watson H., Mitra S., Croden F.C., Taylor M., Wood H.M., Perry S.L., Spencer J.A., Quirke P., Toogood G.J., Lawton C.L. (2018). A Randomised Trial of the Effect of Omega-3 Polyunsaturated Fatty Acid Supplements on the Human Intestinal Microbiota. Gut.

[101] Chakravarthy S.K., Jayasudha R., Prashanthi G.S., Ali M.H., Sharma S., Tyagi M., Shivaji S. (2018). Dysbiosis in the Gut Bacterial Microbiome of Patients with Uveitis, an Inflammatory Disease of the Eye. Indian J. Microbiol..

[102] Tsigalou C., Stavropoulou E., Bezirtzoglou E. (2018). Current Insights in Microbiome Shifts in Sjogren’s Syndrome and Possible Therapeutic Interventions. Front. Immunol..

[103] Weiss E., Katta R. (2017). Diet and Rosacea: The Role of Dietary Change in the Management of Rosacea. Dermatol. Pract. Concept..

[104] Miljanović B., Trivedi K.A., Dana M.R., Gilbard J.P., Buring J.E., Schaumberg D.A. (2005). Relation Between Dietary n−3 and n−6 Fatty Acids and Clinically Diagnosed Dry Eye Syndrome in Women. Am. J. Clin. Nutr..

[105] Maguire M.G., Pistilli M., Ying G.S., Szczotka-Flynn L.B., Hardten D.R., Lin M.C., Shtein R.M. (2018). The Dry Eye Assessment and Management Study Research, n−3 Fatty Acid Supplementation for the Treatment of Dry Eye Disease. N. Engl. J. Med..

[106] Yazdi F.G., Soleimanian-Zad S., van den Worm E., Folkerts G. (2019). Turmeric Extract: Potential Use as a Prebiotic and Anti-Inflammatory Compound?. Plant Foods Hum. Nutr..

[107] Tan Y.F., Li H.L., Lai W.Y., Zhang J.Q. (2013). Crude Dietary Polysaccharide Fraction Isolated from Jackfruit Enhances Immune System Activity in Mice. J. Med. Food.

[108] Thornton S.P., Troyer E. (2006). Treatment for Dry Eye Syndrome. Google Patents: 2006. U.S. Patent.

[109] Chen M., Hu D.N., Pan Z., Lu C.W., Xue C.Y., Aass I. (2010). Curcumin Protects against Hyperosmoticity-Induced Il-1beta Elevation in Human Corneal Epithelial Cell Via MAPK Pathways. Exp. Eye Res..

[110] Chung S.-H., Choi S.H., Choi J.A., Chuck R.S., Joo C.-K. (2012). Curcumin Suppresses Ovalbumin-Induced Allergic Conjunctivitis. Mol. Vis..

[111] Chisari G., Chisari E.M., Francaviglia A., Chisari C.G. (2017). The Mixture of Bifidobacterium Associated with Fructo-Oligosaccharides Reduces the Damage of the Ocular Surface. Clin. Ter..

[112] Hann K., Lentz L. (2006). Synergic Combination of Compositions Containing Aloe Vera Isolates and Their Therapeutic Application. Google Patents: 2006. U.S. Patent.

[113] Bode L. (2012). Human Milk Oligosaccharides: Every Baby Needs a Sugar Mama. Glycobiology.

[114] Chaumeil C., Liotet S., Kogbe O., Sullivan D.A. (1994). Treatment of Severe Eye Dryness and Problematic Eye Lesions with Enriched Bovine Colostrum Lactoserum. Lacrimal Gland, Tear Film, and Dry Eye Syndromes: Basic Science and Clinical Relevance.

[115] Bucolo C., Musumeci M., Salomone S., Romano G.L., Leggio G.M., Gagliano C., Reibaldi M., Avitabile T., Uva M.G., Musumeci S. (2015). Effects of Topical Fucosyl-Lactose, a Milk Oligosaccharide, on Dry Eye Model: An Example of Nutraceutical Candidate. Front. Pharmacol..

[116] Diego J.L., Bidikov L., Pedler M.G., Kennedy J.B., Quiroz-Mercado H., Gregory D.G., Petrash J.M., McCourt E.A. (2016). Effect of Human Milk as a Treatment for Dry Eye Syndrome in a Mouse Model. Mol. Vis..

[117] Lam T.K., Shao S., Zhao Y., Marincola F., Pesatori A., Bertazzi P.A., Caporaso N.E., Wang E., Landi M.T. (2012). Influence of Quercetin-Rich Food Intake on Microrna Expression in Lung Cancer Tissues. Cancer Epidemiol. Biomark. Prev..

[118] McKay T.B., Karamichos D. (2017). Quercetin and the Ocular Surface: What We Know and Where We are Going. Exp. Biol. Med..

[119] Abengozar-Vela A., Calonge M., Stern M.E., Gonzalez-Garcia M.J., Enriquez-De-Salamanca A. (2015). Quercetin and Resveratrol Decrease the Inflammatory and Oxidative Responses in Human Ocular Surface Epithelial Cells. Investig. Ophthalmol. Vis. Sci..

[120] Abengozar-Vela A., Schaumburg C.S., Stern M.E., Calonge M., Enriquez-de-Salamanca A., Gonzalez-Garcia M.J. (2018). Topical Quercetin and Resveratrol Protect the Ocular Surface in Experimental Dry Eye Disease. Ocul. Immunol. Inflamm..

[121] Oh H.N., Kim C.E., Lee J.H., Yang J.W. (2015). Effects of Quercetin in a Mouse Model of Experimental Dry Eye. Cornea.

[122] Bindels L.B., Delzenne N.M., Cani P.D., Walter J. (2015). Towards a More Comprehensive Concept for Prebiotics. Nat. Rev. Gastroenterol. Hepatol..

[123] Lilly D.M., Stillwell R.H. (1965). Probiotics: Growth-Promoting Factors Produced by Microorganisms. Science.

[124] Mackowiak P. (2013). Recycling Metchnikoff: Probiotics, the Intestinal Microbiome and the Quest for Long Life. Front. Public Health.

[125] Bron P.A., van Baarlen P., Kleerebezem M. (2011). Emerging Molecular Insights into the Interaction Between Probiotics and the Host Intestinal Mucosa. Nat. Rev. Microbiol..

[126] Foligné B., Daniel C., Pot B. (2013). Probiotics from Research to Market: The Possibilities, Risks and Challenges. Curr. Opin. Microbiol..

[127] Gareau M.G., Sherman P.M., Walker W.A. (2010). Probiotics and the Gut Microbiota in Intestinal Health and Disease. Nat. Rev. Gastroenterol. Hepatol..

[128] Sokol H., Pigneur B., Watterlot L., Lakhdari O., Bermúdez-Humarán L.G., Gratadoux J.-J., Blugeon S., Bridonneau C., Furet J.-P., Corthier G. (2008). Faecalibacterium Prausnitzii Is an Anti-Inflammatory Commensal Bacterium Identified by Gut Microbiota Analysis of Crohn Disease Patients. Proc. Natl. Acad. Sci. USA.

[129] Yahfoufi N., Mallet J.F., Graham E., Matar C. (2018). Role of Probiotics and Prebiotics in Immunomodulation. Curr. Opin. Food Sci..

[130] Brenner D.M., Moeller M.J., Chey W.D., Schoenfeld P.S. (2009). The Utility of Probiotics in the Treatment of Irritable Bowel Syndrome: A Systematic Review. Am. J. Gastroenterol..

[131] Hoveyda N., Heneghan C., Mahtani K.R., Perera R., Roberts N., Glasziou P. (2009). A Systematic Review and Meta-Analysis: Probiotics in the Treatment of Irritable Bowel Syndrome. BMC Gastroenterol..

[132] McFarland L.V., Dublin S. (2008). Meta-Analysis of Probiotics for the Treatment of Irritable Bowel Syndrome. World J. Gastroenterol..

[133] Moayyedi P., Ford A.C., Talley N.J., Cremonini F., Foxx-Orenstein A.E., Brandt L.J., Quigley E.M.M. (2010). The Efficacy of Probiotics in the Treatment of Irritable Bowel Syndrome: A Systematic Review. Gut.

[134] Ritchie M.L., Romanuk T.N. (2012). A Meta-Analysis of Probiotic Efficacy for Gastrointestinal Diseases. PLoS ONE.

[135] Whelan K. (2011). Probiotics and Prebiotics in the Management of Irritable Bowel Syndrome: A Review of Recent Clinical Trials and Systematic Reviews. Curr. Opin. Clin. Nutr. Metab. Care.

[136] Whelan K., Quigley E.M. (2013). Probiotics in the Management of Irritable Bowel Syndrome and Inflammatory Bowel Disease. Curr. Opin. Gastroenterol..

[137] Holubar S.D., Cima R.R., Sandborn W.J., Pardi D.S. (2010). Treatment and Prevention of Pouchitis after Ileal Pouch-Anal Anastomosis for Chronic Ulcerative Colitis. Cochrane Database Syst. Rev..

[138] Shen J., Zuo Z.-X., Mao A.-P. (2013). Effect of Probiotics on Inducing Remission and Maintaining Therapy in Ulcerative Colitis, Crohn’s Disease, and Pouchitis: Meta-analysis of Randomized Controlled Trials. Inflamm. Bowel Dis..

[139] Reading R. (2001). Probiotics in Primary Prevention of Atopic Disease: A Randomised Placebo-Controlled Trial. Ambul. Child Health.

[140] Ohland C.L., MacNaughton W.K. (2010). Probiotic Bacteria and Intestinal Epithelial Barrier Function. Am. J. Physiol. Gastrointest. Liver Physiol..

[141] Ng S.C., Hart A.L., Kamm M.A., Stagg A.J., Knight S.C. (2008). Mechanisms of Action of Probiotics: Recent Advances. Inflamm. Bowel Dis..

[142] Lee A., Lee Y.J., Yoo H.J., Kim M., Chang Y., Lee D.S., Lee J.H. (2017). Consumption of Dairy Yogurt Containing Lactobacillus paracasei ssp. paracasei, Bifidobacterium animalis ssp. lactis and Heat-Treated Lactobacillus plantarum Improves Immune Function Including Natural Killer Cell Activity. Nutrients.

[143] Zhang M., Zhang S., Hua Z., Zou X. (2017). Long-Term Use of Bifidobacterium Longum Alleviates Colorectal Colitis in Rats by Regulating Inflammatory Cytokines and Treg Cells. Int. J. Clin. Exp. Med..

[144] Peng G.-C., Hsu C.-H. (2005). The Efficacy and Safety of Heat-Killed Lactobacillus Paracasei for Treatment of Perennial Allergic Rhinitis Induced by House-Dust Mite. Pediatric Allergy Immunol..

[145] Perdigon G., Fuller R., Raya R. (2001). Lactic Acid Bacteria and Their Effect on the Immune System. Curr. Issues Intest. Microbiol..

[146] Tanabe S. (2013). The Effect of Probiotics and Gut Microbiota on Th17 Cells. Int. Rev. Immunol..

[147] Güvenç I.A., Muluk N.B., Mutlu F.Ş., Eşki E., Altıntoprak N., Oktemer T., Cingi C. (2016). Do Probiotics Have a Role in the Treatment of Allergic Rhinitis? A Comprehensive Systematic Review and Metaanalysis. Am. J. Rhinol. Allergy.

[148] Jayasudha R., Kalyana Chakravarthy S., Sai Prashanthi G., Sharma S., Garg P., Murthy S.I., Shivaji S. (2018). Alterations in Gut Bacterial and Fungal Microbiomes Are Associated with Bacterial Keratitis, an Inflammatory Disease of the Human Eye. J. Biosci..

[149] Szymula A., Rosenthal J., Szczerba B.M., Bagavant H., Fu S.M., Deshmukh U.S. (2014). T Cell Epitope Mimicry Between Sjögren’s Syndrome Antigen A (SSA)/Ro60 and Oral, Gut, Skin and Vaginal Bacteria. Clin. Immunol..

[150] Kim J., Choi H.S., Kim J.Y., Jeong J.H., Ryu S.J., Lee J.H., Kim W.T., Im S.-H., Oh Y.J., Kim K.M. (2017). Clinical Effect of IRT-5 Probiotics on Immune Modulation of Autoimmunity or Alloimmunity in the Eye. Nutrients.

[151] Poussier P., Ning T., Banerjee D., Julius M. (2002). A Unique Subset of Self-specific Intraintestinal T Cells Maintains Gut Integrity. J. Exp. Med..

[152] Chisari G., Rampello L., Chisari E.M., Catania V.E., Greco C., Stagni E., Chisari C.G. (2016). Microbiology Synergism Between Tear Substitutes and Symbiotic Treatment of Patients with Irritable Bowel Syndrome. Acta Med. Mediterr..

[153] Kawashima M., Nakamura S., Izuta Y., Inoue S., Tsubota K. (2016). Dietary Supplementation with a Combination of Lactoferrin, Fish Oil, and Enterococcus faecium WB2000 for Treating Dry Eye: A Rat Model and Human Clinical Study. Ocul. Surf..

[154] Chisari G., Chisari E.M., Borzi A.M., Chisari C.G. (2017). Aging Eye Microbiota in Dry Eye Syndrome in Patients Treated with Enterococcus faecium and Saccharomyces boulardii. Curr. Clin. Pharmacol..

[155] Wang T.H., Anvari S., Anagnostou K. (2019). The Role of Probiotics in Preventing Allergic Disease. Children.

[156] Fechtner R.D., Godfrey D.G., Budenz D., Stewart J.A., Stewart W.C., Jasek M.C. (2010). Prevalence of Ocular Surface Complaints in Patients with Glaucoma Using Topical Intraocular Pressure-Lowering Medications. Cornea.

[157] Garcia-Feijoo J., Sampaolesi J.R. (2012). A Multicenter Evaluation of Ocular Surface Disease Prevalence in Patients with Glaucoma. Clin. Ophthalmol..

[158] Bae H.W., Kim J.H., Kim S., Kim M., Lee N., Hong S., Seong G.J., Kim C.Y. (2015). Effect of Korean Red Ginseng Supplementation on Dry Eye Syndrome in Glaucoma Patients–A randomized, Double-Blind, Placebo-Controlled Study. J. Ginseng Res..

[159] Kim N.-R., Kim J.-H., Kim C.-Y. (2010). Effect of Korean Red Ginseng Supplementation on Ocular Blood Flow in Patients with Glaucoma. J. Ginseng Res..

[160] Ramsay E.I., Rao S., Madathil L., Hegde S.K., Baliga-Rao M.P., George T., Baliga M.S. (2019). Honey in Oral Health and Care: A Mini Review. J. Oral Biosci..

[161] Yaghoobi R., Kazerouni A., Kazerouni O. (2013). Evidence for Clinical Use of Honey in Wound Healing as an Anti-bacterial, Anti-inflammatory Anti-oxidant and Anti-viral Agent: A Review. Jundishapur J. Nat. Pharm. Prod..

[162] Fragiadakis G.K., Smits S.A., Sonnenburg E.D., Van Treuren W., Reid G., Knight R., Manjurano A., Changalucha J., Dominguez-Bello M.G., Leach J. (2019). Links Between Environment, Diet, and the Hunter-Gatherer Microbiome. Gut Microbes.

[163] George Kerry R., Patra J.K., Gouda S., Park Y., Shin H.-S., Das G. (2018). Benefaction of Probiotics for Human Health: A review. J. Food Drug Anal..

[164] Mazruei Arani N., Emam-Djomeh Z., Tavakolipour H., Sharafati-Chaleshtori R., Soleimani A., Asemi Z. (2018). The Effects of Probiotic Honey Consumption on Metabolic Status in Patients with Diabetic Nephropathy: A Randomized, Double-Blind, Controlled Trial. Probiotics Antimicrob. Proteins.

[165] Molan P.C. (1999). Why Honey is Effective as a Medicine. 1. Its Use in Modern Medicine. Bee World.

[166] Ajibola A. (2015). Novel Insights into the Health Importance of Natural Honey. Malays. J. Med. Sci..

[167] Cernak M., Majtanova N., Cernak A., Majtan J. (2012). Honey Prophylaxis Reduces the Risk of Endophthalmitis During Perioperative Period of Eye Surgery. Phytother. Res..

[168] Albietz J.M., Lenton L.M. (2015). Standardised Antibacterial Manuka Honey in the Management of Persistent Post-Operative Corneal Oedema: A Case Series. Clin. Exp. Optom..

[169] Jankauskiene J., Jarushaitiene D., Cheksteryte V., Rachys J. (2007). Using 20% Honey Solution Eye Drops in Patients with Dry Eye Syndrome. J. Apic. Res..

[170] Albietz J.M., Schmid K.L. (2017). Randomised Controlled Trial of Topical Antibacterial Manuka (Leptospermum Species) Honey for Evaporative Dry Eye due to Meibomian Gland Dysfunction. Clin. Exp. Optom..

[171] Ghosh D., Konishi T. (2007). Anthocyanins and Anthocyanin-Rich Extracts: Role in Diabetes and Eye Function. Asia Pac. J. Clin. Nutr..

[172] Riva A., Togni S., Franceschi F., Kawada S., Inaba Y., Eggenhoffner R., Giacomelli L. (2017). The Effect of a Natural, Standardized Bilberry Extract (Mirtoselect^®^) in Dry Eye: A Randomized, Double Blinded, Placebo-Controlled Trial. Eur. Rev. Med. Pharmacol. Sci..

[173] Masmali A.M., Alanazi S.A., Alotaibi A.G., Fagehi R., Abusharaha A., El-Hiti G.A. (2019). The Acute Effect of a Single Dose of Green Tea on the Quality and Quantity of Tears in Normal Eye Subjects. Clin. Ophthalmol..

[174] Nejabat M., Reza S.A., Zadmehr M., Yasemi M., Sobhani Z. (2017). Efficacy of Green Tea Extract for Treatment of Dry Eye and Meibomian Gland Dysfunction; A Double-blind Randomized Controlled Clinical Trial Study. J. Clin. Diagn. Res..

[175] Song J.-M., Lee K.-H., Seong B.-L. (2005). Antiviral Effect of Catechins in Green Tea on Influenza Virus. Antivir. Res..

[176] Forester S.C., Lambert J.D. (2011). The Role of Antioxidant Versus Pro-Oxidant Effects of Green Tea Polyphenols in Cancer Prevention. Mol. Nutr. Food Res..

[177] Rains T.M., Agarwal S., Maki K.C. (2011). Antiobesity Effects of Green Tea Catechins: A Mechanistic Review. J. Nutr. Biochem..

[178] Natarajan S.B., Hwang J.-W., Kim Y.-S., Kim E.-K., Park P.-J. (2017). Ocular Promoting Activity of Grape Polyphenols—A Review. Environ. Toxicol. Pharmacol..

[179] Baydar N.G., Özkan G., Sağdiç O. (2004). Total Phenolic Contents and Antibacterial Activities of Grape (*Vitis vinifera* L.) Extracts. Food Control.

[180] Kokke K.H., Morris J.A., Lawrenson J.G. (2008). Oral Omega-6 Essential Fatty Acid Treatment in Contact Lens Associated Dry Eye. Contact Lens Anterior Eye.

[181] Lieberman S., Enig M.G., Preuss H.G. (2006). A Review of Monolaurin and Lauric Acid: Natural Virucidal and Bactericidal Agents. Altern. Complement. Ther..

[182] Lin Y.-C., Schlievert P.M., Anderson M.J., Fair C.L., Schaefers M.M., Muthyala R., Peterson M.L. (2009). Glycerol Monolaurate and Dodecylglycerol Effects on Staphylococcus aureus and Toxic Shock Syndrome Toxin-1 In Vitro and In Vivo. PLoS ONE.

[183] Zhu H., Flanagan J.L. (2019). Compositions, Methods and/or Devices for Prevention and/or Treatment of Dry Eye Disorders. Google Patents: 2019. U.S. Patent.

[184] Holland K.T., Taylor D., Farrell A.M. (1994). The Effect of Glycerol Monolaurate on Growth of, and Production of Toxic Shock Syndrome Toxin-1 and Lipase by, Staphylococcus aureus. J. Antimicrob. Chemother..

[185] Peterson M.L., Schlievert P.M. (2006). Glycerol Monolaurate Inhibits the Effects of Gram-Positive Select Agents on Eukaryotic Cells. Biochemistry.

[186] Palmieri B., Destefanis S., Giretto D., Muscolo C., Di Cerbo A., Guidetti G., Canello S. An Intriguing Nutraceutical Approach in Dogs Affected by Keratoconjunctivitis Sicca. Proceedings of the 4th International Conference on Clinical and Experimental Ophthalmology.

